# Concomitant Imperforate Hymen and Transverse Vaginal Septum Complicated with Pyocolpos and Abdominovaginal Fistula

**DOI:** 10.1155/2014/406219

**Published:** 2014-02-10

**Authors:** Berna Dilbaz, Sadiman Kiykac Altinbas, Namik Kemal Altinbas, Ozlem Sengul, Serdar Dilbaz

**Affiliations:** ^1^Department of Obstetrics and Gynecology, Etlik Zubeyde Hanim Women's Health Training and Research Hospital, Ankara, Turkey; ^2^Department of Radiology, Diskapi Children's Hospital and Hematology Oncology Education and Research Hospital, Ankara, Turkey

## Abstract

A 13-year-old patient with a complaint of worsening lower abdominal pain during the past 4 months was admitted to the emergency department. An abdominopelvic ultrasound scan revealed a distended uterocervical cavity suggestive of hematometrocolpos. Imperforate hymen was observed on examination of the external genitalia. MRI scan revealed an air-fluid level representing pyometrocolpos within a distended vagina. Posterior vaginal extraperitoneal leakage as the sign of a fistula between the vagina and the rectovaginal space was detected. Although laparoscopic approach was planned, malodorous pus expelled after the insertion of the Veress needle, it was decided to proceed to laparotomy. Pus with peritoneal microabscess formations was observed at laparotomy. The imperforate hymen and TVS were excised vaginally. A more complex anomaly should be suspected in cases with hematometra and concomitant imperforated hymen without any bulging and thorough evaluation using radiological imaging techniques should be performed before surgical approach.

## 1. Introduction

The prevalence of Mullerian duct anomalies, although rare, ranges from 0.001% to 10% in general population and 8–10% in women with an adverse reproductive history [1]. Among these anomalies, imperforate hymen is the most common anomaly with an incidence of 1/1,000 to 1/10,000 [2]. Transverse vaginal septum (TVS) with a prevalence of 1 in 30,000 to 1 in 80,000 women is more rare than imperforate hymen [3]. Both obstructive vaginal malformations may be asymptomatic in childhood or become symptomatic due to mucocolpos with the stimulation of maternal estradiol. Most of the cases become symptomatic after menarche with a complaint of hematometrocolpos. Concomitant imperforate hymen and TVS with unicornuate uterus [4] and concomitant imperforate hymen with a uterocervicovaginal septum [5] were presented previously. Congenital vesicovaginal [6] and iatrogenic or congenital urethrovaginal fistula formations [7, 8] due to obstructive vaginal lesions have been described, but the current case is the first to have abdominovaginal fistula related to both imperforate hymen and TVS.

## 2. Case Report

A 13-year-old premenarchal girl was admitted to the emergency department with a complaint of worsening lower abdominal pain during the past 4 months. Secondary sexual characteristics had developed normally; she had Tanner stage IV breasts and normal pubic and axillary hair development compatible with her age.

**Figure 1:**
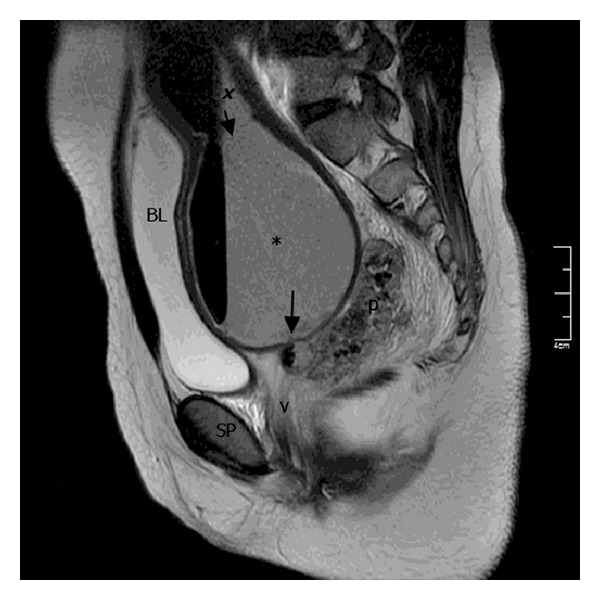
MR imaging demonstrating pus within a distended vagina, endometrial and endocervical canal, and posterior vaginal extraperitoneal leakage (p). A complete mid-transverse vaginal septum was confirmed surgically. Sagittal T2-weighted fast spin echo (FSE) image shows distention of the endometrial (*x*), endocervical (short arrow), and vaginal (*) canals by low-signal-intensity material with air-fluid level representing pus. Long arrow is pointing at a fistula tract (a collection) between the vagina and rectum. The level of the obstruction lies above the symphysis pubis (SP).

**Figure 2:**
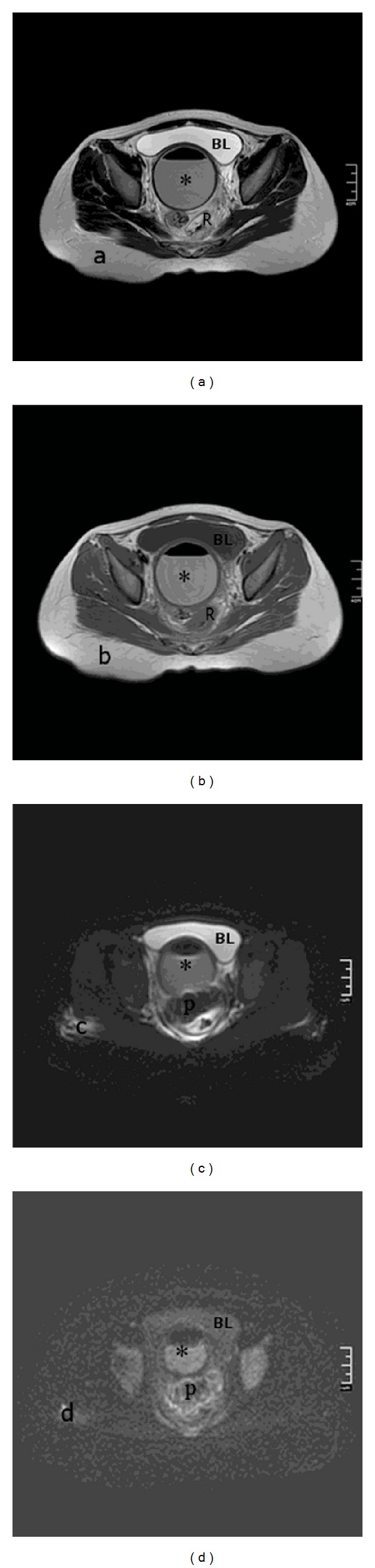
(a) Axial T2-weighted FSE image. Material with air-fluid level shows lower signal-intensity than bladder (BL). (b) Axial T1-weighted FSE image. Material with air-fluid level shows higher signal-intensity than bladder (BL), ((c), (d)) apparent diffusion coefficient (ADC), and diffusion-weighted image (DWI). Restricted diffusion indicates purulent collection in the rectovaginal space, pus (P), and vagina (*) rectum was compressed by the rectovaginal space leakage posterolaterally, rectum (R).

The absence of a hymeneal opening without any bulging was observed on examination of the external genitalia. The abdomen was tender and distended. At admission, her laboratory investigations were within normal limits with a leucocyte count of 5,800/mm^3^ and C-reactive proteins were low. An abdominopelvic ultrasound scan revealed a distended uterocervical cavity suggestive of hematometrocolpos compressing the urinary bladder without any other pathology of the genitourinary system. For a thorough evaluation, the patient was taken to the operating theater to be examined under anesthesia. After rectal examination, the presence of imperforate hymen was confirmed and a tender soft mass in the upper part of the vagina was detected. The imperforate hymen was excised and a short vaginal blind pouch of 2 cm bearing vaginal rugae was visualized. As the incision did not result in drainage of the collection, presence of a thick TVS was suspected. The patient was scheduled for a magnetic resonance imaging (MRI) scan. While waiting for her appointment for MRI scan at the hospital, the patient had a worsening abdominal pain with vomiting and her leucocyte count was elevated from 5800/mm^3^ first to 13,000/mm^3^ and within 6 hours reached to 15,000/mm^3^. There was only 24 hours between trial of hymenectomy and MRI scan. MRI scan revealed an air-fluid level representing pyometrocolpos within a distended vagina, endometrial cavity, and endocervical canal. Posterior vaginal extraperitoneal leakage was detected and it was interpreted as the sign of a fistula (long arrow) between the vagina and the rectovaginal space (Figure 1). The level of the obstruction was suspected to be on the middle third of the vagina and the rectum was compressed by the leakage filling the rectovaginal space posterolaterally (Figure 2). Although laparoscopic approach was planned, malodorous pus expelled after the insertion of the Veress needle, it was decided to proceed to laparotomy. At laparotomy, pus with peritoneal microabscess formations was observed. The pus was sampled for culture and antibiogram and drained followed by intra-abdominal washing. Escherichia coli grew from the culture; both taken during the operation from the pus found in the abdominal cavity and the specimen taken from the drained pus.

**Figure 3:**
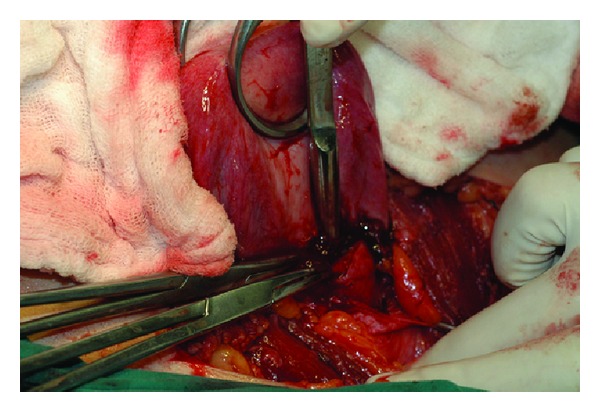
The guide of a number 8 Hegar dilator inserted from the incision site to localize the transverse vaginal septum. The figure also shows uterus and bilateral tubes normal in size and shape for the age of the patient.

The uterus and bilateral tubes were normal in size and shape for the age of the patient; they were pink and fresh looking. A close observation of the uterus and cervix revealed no possible perforation sites that would explain intraabdominal spillage of the pus. A general surgeon was invited to the operation to rule out a probable rectal fistula but neither a fistula nor a perforation site was observed. As the probable communication or the tract between the abdomen and vagina was not observed, it could not be resected or repaired. In order to provide the drainage of pyocolpos, a 1 cm transverse incision at the superior part of the lower uterine segment was performed and with the guide of a number 8 Hegar dilator that was inserted from the incision site and then passed through the cervix into the vagina blindly, the septum was localized (Figure 3). Then the TVS was excised vaginally, with the guidance of the Hegar dilator. Almost 200 mL of dense, brownish, malodorous pus was drained with suction. After total excision of the TVS, the cervix became clearly visible at the upper part of the vagina. A Foley sound was inserted into the cavity for the drainage of the collection and the uterine incision was repaired. A silicon drain was placed at the pouch of Douglas before the closure of the abdominal wall. The operation completed uneventfully. During the postoperative follow-up, pus drainage continued both from the silicon drain and via the posterior vaginal fornix. The patient received appropriate antibiotics regimens according to the culture results. The patient was scheduled for a control MRI after cessation of the drainage of the pus from the drains. Repeated MRI confirmed complete resolution of the pelvic collection. The patient was discharged uneventfully. She had normal regular menstrual cycles without any pain and complaints during the 8 months that she was followed after the surgery. The patient used a vaginal dilator to prevent stenosis for two months after the operation and clinical evaluations at 4, 6, and 8 months postoperatively confirmed an adequate vaginal space without any stricture.

## 3. Discussion

Various congenital anomalies of the female tract such as agenesis, failure of vertical or lateral fusion, and failure of canalization occur when normal development of Mullerian duct disrupts in any stage of the developmental milestones. The caudal ends fuse in the midline and are occluded by the Mullerian tubercle that forms the hymen. Failure of this step with degeneration of the epithelial plate results in imperforate hymen. A transverse vaginal septum results from a defect of incomplete vertical fusion between Mullerian tubercle and urogenital sinus with failure of canalization of the vaginal plate [4]. With current data, it is still under debate whichever mechanisms induce the issue.

In the current case, a transverse vaginal septum and imperforate hymen were detected and the level of TVS was the middle third of the vagina. When hematometrocolpos was firstly diagnosed, clinical presentation of the patient seemed to be restricted to an obstructive pathology only. The pathology was complicated with infection and development of pyometrocolpos with a fistula between the vagina and the rectovaginal space. There was only 24 hours between trial of hymenectomy and the laparotomy that revealed the presence of abscess, so the abscess formation was not thought to be related with the initial procedure. The mechanism of fistula formation was not clear due to its unique nature. As the patient was 13 years old and she had the onset of complaints 4 months ago, presumably her menarche began only 4 months ago and accumulation of the menstrual blood and causing the described entity took months. We are not sure whether this duration is enough for formation of a chronic fistula with an epithelialized tract. As this was not present, it might well be due to spontaneous rupture of the abscess. Therefore, we addressed the probable mechanism that might explain the formation of the fistula or the tract as increased intracavitary pressure due to gradually accumulating collection of the infectious material and failure of drainage because of the resistance of thick TVS. The probable mechanism for Escherichia coli growth from the culture was thought to be related to the close proximity to the bowel.

Although it is well known that hematometra is a good medium for infectious development, occurrence of severe, extensive infection in the abdomen with a healthy looking uterus and tubes in this case was hard to explain.

To our knowledge, the present case is the first reported case of a fistula formation between posterior fornix and rectovaginal space associated with pyocolpos and obstructive multiple Mullerian anomalies. A more complex anomaly should be suspected in cases with hematometra and concomitant imperforated hymen without any bulging and thorough evaluation using radiological imaging techniques should be performed before surgical approach. In these pathologies, other female reproductive tract anomalies and abnormalities of the renal and skeletal systems are often related because of the embryogenetic origin. The evaluation of these anomalies should be done regarding these coexisting defects and the right treatment should be planned after defining the anatomy of the pathology as definitely as possible with proper imaging techniques such as an ultrasound scan and MRI.
